# From nonintubation to tubeless: redefining perioperative strategies in modern anesthesiology and thoracic surgery

**DOI:** 10.1097/MS9.0000000000004252

**Published:** 2025-11-04

**Authors:** Ran Zhong, Zihao Liu, Hui Liu, Eugenio Pompeo, Diego Gonzalez-Rivas, Paolo A. Ferrari, Bassam Redwan, Alberto Aiolfi, Luca Bertolaccini, Gabor Kiss, Keng-Leong Ang, Kyung Soo Kim, Jianxing He

**Affiliations:** a Department of Thoracic Surgery and Oncology, The Key Laboratory of Advanced Interdisciplinary Studies, National Center for Respiratory Medicine, National Clinical Research Center for Respiratory Disease, State Key Laboratory of Respiratory Disease, Guangzhou Institute of Respiratory Health, The First Affiliated Hospital of Guangzhou Medical University, Guangzhou, China; b Department of Anesthesiology, The Key Laboratory of Advanced Interdisciplinary Studies, National Center for Respiratory Medicine, National Clinical Research Center for Respiratory Disease, State Key Laboratory of Respiratory Disease, Guangzhou Institute of Respiratory Health, The First Affiliated Hospital of Guangzhou Medical University, Guangzhou, China; c Unit of Thoracic Surgery, Department of Surgical Sciences, University of Rome Tor Vergata, Rome, Italy; d Division of Thoracic Surgery, University Hospital Policlinico Tor Vergata, Rome, Italy; e Department of Thoracic Surgery and Minimally Invasive Thoracic Surgery Unit (UCTMI), Coruña University Hospital, Coruña, Spain; f Department of Thoracic Surgery and Interventional Endoscopy, A.R.N.A.S. “G. Brotzu”, Cagliari, Italy; g Department of Thoracic Surgery, Knappschaft Kliniken Lünen, Lünen, Germany; h University of Witten/Herdecke, Witten, Germany; i I.R.C.C.S. Ospedale Galeazzi – Sant’Ambrogio, Division of General Surgery, Department of Biomedical Science for Health, University of Milan, Milan, Italy; j Department of Thoracic Surgery, IEO European Institute of Oncology IRCCS, Milan, Italy; k University Hospital of Saint Denis, Reunion Island, France; l Department of Thoracic Surgery, Freeman Hospital, Newcastle upon Tyne, United Kingdom; m Department of Thoracic and Cardiovascular Surgery, Seoul St. Mary’s Hospital, College of Medicine, The Catholic University of Korea, Seoul, Republic of Korea

## The emergence of “tubeless”

Tubeless thoracic anesthesia, which involves the elimination of unnecessary catheters and without or avoiding endotracheal intubation during thoracic surgery while maintaining spontaneous breathing, has been incorporated into *Cohen’s Comprehensive Thoracic Anesthesia*, an authoritative work edited by Edmond Cohen. This inclusion marks a pivotal academic milestone, elevating “tubeless” from a clinical innovation to a widely accepted and widely promoted technique in modern thoracic anesthesia.

The term “tubeless” signifies a transformative paradigm shift in perioperative medicine, advancing beyond the conventional framework of intubation to nonintubation approaches, without the need of invasive tubes, such as chest tube, urinary catheter. This concept does not propose its application for all cases; instead, it promotes the avoidance of any invasive tubes whenever possible, based on patient’s clinical situations, supported by evidence-based medicine and clinical practice guidelines. Even if these measures can reduce iatrogenic harm to patients by 10% or less, they represent significant clinical value and a meaningful contribution to improving patient outcomes.

In clinical practice, this philosophy is achieved through a highly individualized and evidence-based approach. It involves carefully evaluating the necessity of endotracheal intubation based on real-time patient-specific parameters; rigorously managing the need and duration of urinary catheterization; selectively using chest drains with a focus on early removal, and exercising prudence in the application of nasogastric tubes and other invasive monitoring techniques. A key feature of this approach is its operational flexibility, allowing real-time adjustments in airway management and catheter use based on patient condition and surgical demands. Temporary intubation may be justified in certain circumstances, such as in patients undergoing prolonged surgery, or those with an increased amount of airway secretions. Still, the overarching goal remains to minimize invasiveness of the procedure. This is crucial because excessive invasiveness can trigger detrimental immune responses[1] that may compromise patient recovery and increase the risk of postoperative complications. By adopting this nuanced strategy, healthcare providers can strike a balance between providing necessary medical interventions and safeguarding patients’ physiological integrity.

The shift in terms from “nonintubation” to “tubeless” reflects the adoption of a refined, patient-centric paradigm. The term “tubeless” encapsulates a broader and more sophisticated strategy that integrates temporary intubation when warranted, prioritizes early extubation for critically ill patients who require intubation to shorten mechanical ventilation time, supports transition to less invasive ventilatory modes [like using laryngeal mask airway (LMA)], and encompasses multiple dimensions of minimally invasive care. It involves the dynamic adjustment to patient status throughout the perioperative continuum. This flexibility is especially valuable in contemporary surgical settings, where patient physiology and procedural demands are constantly changing. By replacing the static non-intubation approach (characterized by a single-stage, non-phased, and with unaltered airway tools) with the dynamic tubeless method (characterized by a phased approach that adapts to surgical progression), the application scope of non-intubation techniques can be broadened. This transformation enables these techniques to deliver tangible clinical benefits on a broader scale and facilitates flexibly to diverse range of patient conditions and treatment scenarios. This study was conducted and reported in accordance with the TITAN Guidelines 2025 on the governance of artificial intelligence (AI) in research and medical writing[2].

## Clinical scenarios

In thoracoscopic surgery, the tubeless anesthesia model realizes its practical application through a meticulously stepwise approach[3]. During its inception, the procedure typically begins with a safety-oriented initial orotracheal intubation, which was promptly followed by a transition to a LMA once patient stability was ensured[4]. This approach is further enhanced by the implementation of regional anesthesia techniques, such as thoracic paravertebral blocks, which was aimed at reducing systemic opioid consumption and improving perioperative pain management^[3,5]^. Adequate oxygen levels can be sustained through oxygen supplementation alone, with no requirement for additional invasive interventions or modifications to airway management strategies, thereby emphasizing the adaptability of tubeless anesthesia. Once the safety and confidence of this approach is established, it is slowly introduced to a range of procedure from simple wedge resection to more complex airway reconstruction.

Abdominal laparoscopic interventions represent another area in which tubeless strategies can be beneficial. In these cases, clinical execution involves the use of low-pressure pneumoperitoneum to minimize respiratory burden, as well as an early switch to less invasive ventilatory techniques, in surgery with enhanced recovery after surgery (ERAS^®^) protocols^[6,7]^. This multifaceted approach ensures the maintenance of optimal operative conditions while simultaneously promoting patient comfort and accelerating postoperative recovery.

From the respiratory perspective, tubeless protocols are particularly advantageous for managing acute exacerbations of chronic respiratory conditions. The clinical trajectory emphasizes a prompt transition from invasive to noninvasive ventilation, with high-flow nasal cannula therapy employed as a transitional modality. Standardized weaning protocols are integrated to facilitate a safe and effective withdrawal from ventilatory support, thereby minimizing the duration of invasive intervention without compromising respiratory adequacy[8].

## Why “tubeless” aligns with modern clinical needs

### Precision and personalized medicine

The adoption of tubeless strategies exemplifies the core tenets of precision and personalized medicine via the dynamic tailoring of anesthetic interventions to individual patients’ physiological and surgical requirements. This methodology relies extensively on real-time monitoring and continuous optimization of respiratory management. Key components include assessing respiratory mechanics (plateau pressure, peak pressure, compliance), end-tidal CO_2_ tracking, patient comfort and respiratory effort, and point-of-care ultrasound (evaluating lung water via B-lines, pneumothorax via absent pleural sliding). These tools enable informed decisions that align with the patient’s baseline pulmonary status, comorbidities, surgical duration and complexity, and individual preferences.

Comprehensive preoperative risk stratification is integral to this strategy. It entails airway assessments using validated scoring systems, evaluation of respiratory reserve, consideration of comorbidities, psychological assessment, and analysis of procedural complexity. Intraoperatively, dynamic risk management is maintained through real-time data acquisition, early warning systems for predicting complication, established escalation protocols, and frequent reassessment of patient status. This structured and responsive framework significantly enhances patient safety and procedural success.

### Reduction of iatrogenic harm

Tubeless strategies proactively mitigate risks of iatrogenic harm across mechanical and pharmacological domains. Mechanically, avoiding endotracheal intubation prevents vocal cord trauma, tracheal mucosal injury, and ventilator-induced lung injury, preserving airway integrity and diaphragmatic function^[4,9]^. Pharmacologically, our randomized controlled trial (NCT03016858) demonstrated that compared to mechanical ventilation video-assisted thoracoscopic surgery (MV-VATS), tubeless spontaneous ventilation video-assisted thoracic surgery (SV-VATS) reduces intraoperative opioid exposure by 70% (sufentanil: 11.37 vs 20.92 mg; remifentanil: 269.78 vs 404.96 mg; both *P*-values <0.001) and eliminates neuromuscular blockade (e.g., cis-atracurium)[10]. Clinically, this strategy accelerates recovery, with reduced extubation times [12.28 vs 17.30 min; *P* < 0.001, time from the end of anesthesia (when all anesthetic drugs are stopped) to the removal of airway devices (double-lumen endotracheal tubes or LMA)], a shortened post-anesthesia care unit (PACU) stay (25.43 vs 30.67 min; *P* = 0.02), and 25.5% lower anesthesia costs (297.81 vs 399.81; *P* < 0.001). Enhanced outcomes include improved pain control (visual analog scale score reduction: 41%), elevated patient satisfaction, and faster return to daily activities, aligning with ERAS principles^[5,11–13]^. These findings validate the tubeless strategy as a cost-effective alternative for selected thoracic procedures, balancing mechanical protection, pharmacological optimization, and holistic recovery.

### Alignment with ERAS

The integration of tubeless strategies with ERAS protocols creates a synergistic model that optimizes the entire perioperative sequence. Significant clinical benefits are achieved by minimizing the use of interventional tubing. Key outcomes involve shortened intensive care unit lengths of stay, enabled by early patient mobilization and ambulation. This early mobilization not only helps prevent deep vein thrombosis but also preserves respiratory function, maintains muscular strength and integrity, and promotes regular gastrointestinal activity, thereby contributing to overall improved patient recovery and prognosis.

The reduction in the incidences of ventilator-associated pneumonia, pressure ulcers, and urinary tract infections significantly lowers the related complication rates. Additionally, the pain and loss of dignity caused by drainage tubes can be mitigated, and better nutritional support can be ensured. This strategic alignment enhances recovery trajectories and contributes to superior clinical and patient-reported outcomes.

## Challenges in implementing “tubeless” strategies

### Technical expertise and team coordination

The practical implementation of tubeless strategies hinges upon a high degree of technical proficiency and seamless multidisciplinary collaboration. This process begins with comprehensive preoperative planning, which encompasses joint evaluations by anesthesia and surgical teams, the development of shared intraoperative protocols, the establishment of clear communication frameworks, and the predefinition of escalation criteria.

During surgery, real-time coordination among anesthesiologists, surgeons, and nursing teams is essential and includes the synchronous communication of intraoperative events. Once deep visceral and truncal nerve blocks are completed, satisfactory local analgesia and tissue relaxation can be achieved, enabling a rapid reduction in the administration of intravenous anesthetics and muscle (if necessary). Additionally, these strategies involve adjusting ventilation modes and parameters based on CO_2_ accumulation, changes in SpO_2_, and movement amplitude during spontaneous breathing (in line with procedural requirements), effectively managing the surgical field, and promptly addressing unforeseen complications. Such coordination demands a high level of mutual understanding and operational fluency among team members, which is typically cultivated through extensive experience and targeted training.

Advanced monitoring technologies form the cornerstone of safe tubeless implementation. These include continuous capnography; advanced hemodynamic assessment tools such as PiCCO and Vigileo; point-of-care ultrasound, which is used to examine lung conditions, diaphragmatic movement, and assess cardiac function via transthoracic echocardiography; and neuromuscular monitoring systems, which are applied in patients with myasthenia gravis or those requiring the administration of muscle relaxants. Integration of these technologies with electronic health records (EHRs), the bispectral index, and automated alert systems enhances real-time surveillance and early detection of adverse trends, thereby supporting the flexible and responsive nature of tubeless protocols.

### Defining indications and contraindications

Patient selection is a critical determinant of the success of a tubeless strategy. Ideal candidates are typically classified as American Society of Anesthesiologists (ASA) grades I–II and have regular airway evaluations, robust baseline respiratory function, and a body mass index (BMI) of less than 35 kg/m^2^. Surgical procedures conducive to tubeless implementation generally have a short duration (<3 hours), minimal blood loss, limited fluid shifts, and involvement of only superficial or peripheral anatomical sites.

There are several contraindications to the tubeless strategy. Absolute contraindications include difficult airways (Mallampati grading III–IV) and patients with severely compromised physiological reserve who are unable to tolerate surgical stress without mechanical ventilation support. Patients with advanced cardiovascular diseases are generally considered high-risk; however, selected cases may still benefit from tubeless approaches under expert care and tailored anesthetic strategies. Relative contraindications include severe obesity (BMI > 35 kg/m^2^): while substantial mediastinal shift may disrupt the surgical field, for minor procedures such as lung biopsy wedges, studies have demonstrated that obesity does not interfere with complication rates or other secondary outcomes – supporting its safe use with overweighted patients. Procedural factors that may preclude tubeless use are high airway pressure requirements, anticipation of considerable bleeding, prolonged surgical duration, and the need for complex thoracoscopic interventions. A balanced consideration of these risks and potential benefits is essential to making informed decisions. All risks and their handling methods are summarized in Table 1.

**Table 1:** Risks associated with tubeless thoracic surgery

**Risk**	**Timing of occurrence**	**Management**	**How to avoid**
Difficult airway (Mallampati score III–IV)	Preoperative assessment and intraoperative period	Prepare airway rescue equipment (e.g., video laryngoscope, laryngeal mask), convert to intubated anesthesia if necessary	Strict preoperative airway assessment; avoid tubeless strategy for patients with Mallampati score III–IV
Severe obesity (BMI >35 kg/m^2^)	Preoperative assessment and intraoperative period (may affect respiration and operation)	Enhance respiratory monitoring, adjust body position to improve ventilation if needed, convert to intubation promptly if respiratory depression occurs	Select patients with BMI <35 kg/m^2^ preoperatively
Significant comorbidities	Preoperative and intraoperative period (may exacerbate conditions)	Closely monitor vital signs intraoperatively, manage complications (e.g., cardiovascular events) promptly, terminate tubeless surgery if necessary	Choose patients with ASA grade I–II and no significant comorbidities
Massive bleeding	Intraoperative period	Stop bleeding promptly, replenish blood volume; convert to intubation to ensure airway safety if bleeding is uncontrollable	Select surgeries with expected minimal blood loss
Prolonged surgical duration (>3 hours)	Intraoperative period	Closely monitor the patient’s tolerance; switch to mechanical controlled ventilation via laryngeal mask airway if tolerance is insufficient.	Select cases with estimated surgical duration <3 hours
Contralateral pleural rupture	Intraoperative period	Immediately insert a double-lumen tube in the lateral decubitus position to isolate the affected side and maintain ventilation	Surgeons should carefully dissect lung tissues to avoid iatrogenic injury

ASA, American Society of Anesthesiologists grading; BMI, Body Mass Index.

### Risk management and safety

One of the main risks of tubeless surgery is unforeseen respiratory/anesthetic emergencies during the procedure. Being prepared for such situation is an indispensable aspect of implementing tubeless strategies. Intubation equipment must be readily available, and the anesthetists need to be proficient with intubation techniques applicable in the lateral decubitus position. In addition, backup ventilation systems, emergency medications, and advanced airway management devices – such as fiberoptic bronchoscopes, video laryngoscopes, and video supraglottic airway devices – must be readily accessible. Ongoing training through simulation exercises, clear role assignments, and protocol-driven drills ensures the team can swiftly manage unexpected clinical events[14].

Errors in patient selection or inadequate preparedness can lead to serious complications such as hypercapnic encephalopathy, hypoxemia, respiratory failure, and aspiration events. Preventive measures include comprehensive preoperative screening, adherence to strict inclusion criteria, continuous monitoring, and predefined triggers for intervention. The implementation of these safeguards enhances safety while preserving the adaptive benefits of the tubeless approach.

## Clinical recommendations and future directions

### Phased implementation

The successful integration of tubeless strategies necessitates a methodical, multi-phased approach (Table 2). The first phase involves a comprehensive preoperative evaluation of patient, which involves an in-depth review of the patient’s medical history, a validated airway assessment, respiratory function testing, and an analysis of comorbid conditions and their perioperative implications. Concurrently, the surgical team needs to evaluate the complexity of the procedure, its expected duration, potential complications, and contingency protocols.

**Table 2:** Summary of key activities in phased implementation of tubeless strategies

Phase	Key activities
Preoperative evaluation	In-depth review of the patient’s medical history Validated airway assessment Respiratory function testing Analysis of comorbid conditions and their perioperative implications Surgical planning addressing procedure complexity, expected duration, potential complications, and contingency protocols
Induction phase	Standard monitoring setup Baseline parameter documentation Emergency equipment preparation Structured team briefings
Maintenance phase	Ongoing monitoring of vital signs and respiratory indices Dynamic adjustment of ventilatory strategies Constant interdisciplinary communication
Transition phase	Clinical assessment of the patient’s eligibility for discontinuation of ventilatory support Gradual reduction of respiratory assistance Monitoring of recovery indicators
Recovery phase	Multifaceted approach integrating functional assessment Optimization of pain control Vigilant complication monitoring
Ward care phase	Respiratory function assessment Pain management Vital sign monitoring Early mobilization evaluation Thorough discharge readiness assessment to ensure safe transition from the healthcare setting

The intraoperative phase proceeds through three distinct stages:

- The induction phase focuses on establishing a standard monitoring setup, documenting baseline parameters, preparing emergency equipment, and conducting structured team briefings, prior to the start of tubeless anaesthesia and surgical incision. Once the procedure starts, the focus shifts to maintain adequate deep of anesthesia while maintaining spontaneous ventilation so that surgery can be safely performed.
- The maintenance phase entails ongoing monitoring of vital signs and respiratory indices, dynamic adjustment of ventilatory strategies, and constant interdisciplinary communication.
- The transition phase involves clinical assessment of the patient’s eligibility for discontinuation of supplementary respiratory support, gradual reduction of respiratory assistance, and monitoring of recovery indicators.

Following surgery, postoperative care adheres to a structured framework. The recovery phase initiates with a multifaceted approach, integrating hypnosis, functional assessment, optimization of pain control, vigilant tracking of complications, and evaluation of anesthetic emergence. Once transferred to the ward, the immediate care phase focuses on respiratory function assessment, pain management, vital sign monitoring, and early mobilization evaluation. This comprehensive process culminates in a thorough discharge readiness assessment, ensuring all physiological and functional criteria are met for a safe transition from the healthcare setting.

### Multidisciplinary collaboration

Robust interdisciplinary collaboration is pivotal to tubeless success. Surgeons lead preoperative planning – aligning procedural approaches, equipment selection, and risk mitigation – and maintain intraoperative focus on minimally invasive techniques, efficient workflow, clear communication, and emergency preparedness. Anesthesiologists oversee protocol development, staff training, and quality assurance to ensure standardized care. Surgical nurses coordinate intraoperative support, aiding in equipment management, sterile procedures, and real-time team communication. Ward nurses manage postoperative recovery, monitoring vital signs, facilitating early mobilization, and reinforcing patient education. Respiratory therapists provide specialized expertise across all stages, optimizing respiratory care from preoperative assessment to postoperative monitoring. This integrated model safeguards procedural integrity and patient safety.

### Research priorities

Future advancements in tubeless implementation are closely tied to predictive analytics and AI-driven decision support. Emerging tools include intelligent algorithms for patient selection, individualized risk modeling, and dynamic resource allocation. The integration of EHRs with real-time physiological data will enhance predictive accuracy and streamline decision-making.

Technological innovation also plays a vital role. Areas of focus include refining high-frequency jet ventilation, advancing noninvasive positive pressure techniques, developing novel airway devices, and exploring hybrid non-tracheal intubated ventilatory systems. Enhanced monitoring through wearable sensors (e.g., wearable ultrasound patch), portable imaging tools (e.g., wireless ultrasound and portable pulmonary function monitors), and telemedicine integration further expands the capabilities of tubeless applications.

It is essential to transform the anesthesia management approach, moving away from the traditional static model of maintaining deep anesthesia throughout the entire surgery, and promoting a shift toward permitting spontaneous breathing during certain phases. This involves reducing the intensity of mechanically controlled ventilation with a gradual conversion to an assist-control mode. When the patient’s vital status is stable, this transition should be initiated as early as is feasible to promote better patient recovery and optimize surgical outcomes.

## Conclusions

The shift from a non-intubation approach in thoracic surgery to a tubeless one marks a fundamental transformation in perioperative medicine, one that prioritizes minimizing invasiveness through pragmatic, patient-centered care. By integrating technology, precision medicine, and interdisciplinary teamwork, tubeless strategies enable safe and efficient surgical management. As AI, predictive analytics, and wearable technologies continue to evolve, tubeless strategies are expected to gain boarder implementation across clinical settings. This advancement will further redefine standards in minimally invasive perioperative care, aligning with the principles of ERAS and facilitating more personalized, cost-effective, and outcome-driven surgical management.
